# Exacerbation of Ventilation-Induced Lung Injury and Inflammation in Preterm Lambs by High-Dose Nanoparticles

**DOI:** 10.1038/s41598-017-13113-9

**Published:** 2017-10-31

**Authors:** Ishmael M. Inocencio, Robert J. Bischof, Sue D. Xiang, Valerie A. Zahra, Vy Nguyen, Tammy Lim, Domenic LaRosa, Jade Barbuto, Mary Tolcos, Magdalena Plebanski, Graeme R. Polglase, Timothy J. Moss

**Affiliations:** 1 The Ritchie Centre, Hudson Institute of Medical Research and Department of Obstetrics and Gynaecology, Monash University, Melbourne, Australia; 2 Department of Immunology and Pathology, Central Clinical School, Faculty of Medicine, Nursing and Health Sciences, Alfred Hospital Campus, Monash University, Melbourne, Australia; 3 School of Health and Biomedical Sciences, RMIT University, Bundoora, Australia; 4 The Ritchie Centre, Hudson Institute of Medical Research, Biotechnology Research Laboratories, Department of Physiology, Monash University, Melbourne, Australia

## Abstract

Mechanical ventilation of preterm neonates causes lung inflammation and injury, with potential life-long consequences. Inert 50-nm polystyrene nanoparticles (PS50G) reduce allergic inflammation in the lungs of adult mice. We aimed to confirm the anti-inflammatory effects of PS50G in a sheep asthma model, and investigate the effects of prophylactic administration of PS50G on ventilation-induced lung injury (VILI) in preterm lambs. We assessed lung inflammatory cell infiltration, with and without PS50G, after airway allergen challenge in ewes sensitised to house dust mite. Preterm lambs (0.83 gestation) were delivered by caesarean section for immediate tissue collection (n = 5) or ventilation either with (n = 6) or without (n = 5) prophylactic intra-tracheal administration of PS50G nanoparticles (3% in 2 ml). Ventilation was continued for a total of 2 h before tissue collection for histological and biomolecular assessment of lung injury and inflammation. In ewes with experimental asthma, PS50G decreased eosinophilic infiltration of the lungs. Ventilated preterm lambs showed molecular and histological signs of lung injury and inflammation, which were exacerbated in lambs that received PSG50G. PS50G treatment decreased established inflammation in the lungs of asthmatic sheep. However, prophylactic administration of PSG50 exacerbated ventilation-induced lung injury and lung inflammation in preterm lambs.

## Introduction

Preterm birth is the leading cause of neonatal morbidity and mortality^1,2^, largely as a result of lung disease^3^. Structural and functional immaturity of the preterm lungs often necessitate respiratory resuscitation and mechanical ventilation^4^. Unfortunately, up to 85% of preterm infants resuscitated in the delivery room inadvertently receive high tidal volumes^5^, which can cause lasting lung injury. Studies in lambs show that as few as 6 short, high-volume (V_T_ 35 ml/kg) breaths are sufficient to initiate an inflammatory response^6,7^ that has a persisting effect on the lungs^8^ and may contribute to chronic lung disease, manifesting as bronchopulmonary dysplasia (BPD).

BPD is a severe lung disease that develops in 20% of infants who receive mechanical ventilation^9^. It is characterised by the continued requirement for respiratory support for at least 28 days after birth. The pathological features of BPD include simplified alveolar development and abnormal pulmonary vasculature^10^.

Proinflammatory cytokines (Interleukin (IL) -1β, -6 and -8) and the injury response genes, Early Growth Response Gene 1 (*EGR1*), Cysteine-Rich 61 (*CYR-*6*1*), and Connective Tissue Growth Factor (*CTGF*) are up-regulated in response to ventilation-induced lung injury (VILI)^11,12^. These mediators likely play integral roles in propagating abnormal lung development. The inflammatory cascade initiated by ventilation-induced lung injury represents a potentially modifiable pathway to BPD. However, to date there has been limited success in prevention or attenuation of lung inflammation resulting from the initiation of ventilation of the preterm lungs^11^.

Nanoparticles are characterised as particles of any substance with a diameter less than 100 nm^13^. Compared to larger particles, nanoparticles possess a large surface area-to-volume ratio^13,14^. Surfaces can be designed specifically to alter their circulation, and biochemical characteristics such as stability, solubility, specificity and bioactivity^13,14^. The size of nanoparticles enables specific interactions at molecular, cellular and sub-cellular levels, allowing for delivery to almost every tissues site^15,16^. This technology offers a plethora of potential therapeutic uses of nanoparticles, including their use as vaccine vectors and drug carriers, and for targeted gene therapy^14^.

Ostensibly inert nanoparticles can reduce lung inflammation and injury in a proinflammatory environment^17,18^ or can induce proinflammatory effects within the lungs^19,20^. To date, very few studies have examined the efficacy of nanoparticles for reducing lung inflammation and injury in newborns. There is little known about the response of the preterm lungs to nanoparticle exposure. Such fundamental knowledge is necessary if we are to realise the potential of targeted pulmonary delivery of anti-inflammatory agents using nanoparticles as a new approach to prevention of BPD.

Intra-tracheal administration of PS50G nanoparticles coated in inert glycine resulted in a reduction of lung parenchymal inflammation in an adult mouse asthma model^17,21^. We hypothesised that PS50G would have anti-inflammatory properties, like those seen in the adult mouse asthma model, in larger animals. Thus, we aimed to first confirm the anti-inflammatory effect of PS50G in a sheep model of allergic asthma. We also hypothesized PS50G nanoparticles would modulate the proinflammatory response of the preterm lungs to VILI. Therefore, we aimed to determine the effect of prophylactic administration of PS50G on the pulmonary inflammatory response to mechanical ventilation in preterm lambs.

## Materials and Methods

Experimental protocols were approved by the relevant Monash University animal ethics committee. All methods were carried out in accordance with the relevant guidelines and regulations, as described by the National Health and Medical Research Council of Australia^22^.

### Effect of PS50G administration in a sheep asthma model

Female lambs (4–5 months old; n = 3) sensitized to house dust mite allergen (HDM, *Dermatophagoides pteronyssinus*; CSL Ltd., AUS) were twice administered ‘priming’ airway challenges with HDM at two-week intervals^23,24^. Briefly, for airway allergen challenges, sheep were restrained in a harness and HDM (500 µg in 5 ml saline) was delivered into each of the left caudal and right accessory lobes of the lung via the biopsy port of a fiber-optic endoscope^23,25^. Bronchoalveolar lavage (BAL) samples were collected (using the endoscope) at 48 h after a second airway challenge to assess baseline airway inflammation^23,26^ in the absence of nanoparticle administration.

Twenty-four hours before a third airway allergen challenge two weeks later, PS50G nanoparticles (0.2% beads in 2 ml saline) were administered into the caudal lobe of the left lung using the fibre-optic endoscope as detailed above. Saline (2 ml) was administered into the accessory lobe of the right lung as a control. BAL was collected 48 h after a subsequent HDM challenge, from both the left caudal and right accessory lobes. BAL cells were resuspended in PBS/1%BSA, and cytospots prepared and stained with Wright’s solution (Kwik-Diff; Thermo Electron Corp., USA) for differential leukocyte cell counts.

### Effect of PS50G administration in ventilated preterm lambs

Pregnant ewes bearing twins or triplets at 125 ± 1 (mean ± SD) days of gestation were anaesthetized by intravenous injection of thiopentone sodium (20 mg/kg). Ewes were intubated and anaesthesia was maintained by inhalational isoflurane (1.5–2.5% in oxygen; Bomac Animal Health, NSW, Australia) delivered via positive pressure ventilation.

Preterm lambs were delivered by caesarean section and randomized to one of three groups; (1) Ventilated with PS50G administration (VENT+NANO), (2) Ventilated without PS50G administration (VENT) and (3) Unventilated Controls (UVC). After clamping and cutting of the umbilical cord, lambs in the UVC group were immediately killed (sodium pentobarbitone; >100 mg/kg IV) for tissue collection. Lambs in ventilated groups were dried, weighed and placed under an infant warmer (CosyCot, Fisher and Paykel Healthcare, East Tamaki, New Zealand). Ventilation was initiated, and polyvinyl catheters (ID 0.86 mm, OD 1.52 mm, Dural Plastics, Australia) filled with heparinised saline [10 IU/ml heparin (Multiparin Heparin, Fisons Pty Ltd, Australia)] were inserted into an umbilical artery and vein to monitor mean arterial pressure and maintain anaesthesia (Alfaxane 5–15 mg/kg/h; Jurox, East Tamaki, Auckland, New Zealand), respectively. Anaesthesia was maintained for the duration of the experiment.

After delivery of the last lamb the ewe was immediately killed (sodium pentobarbitone; >100 mg/kg IV) without recovery from anaesthesia.

Ventilation of lambs was initiated with warm, humidified air, a peak inspiratory pressure (PIP) of 40 cmH_2_O and a positive end-expiratory pressure (PEEP) of 0 cmH_2_O (Dräger “Babylog 8000+, Dräger Medical, Lubeck, Germany), to target a high tidal volume (V_T_) of (10–12 ml/kg). PIP was adjusted to a maximum of 50 cmH_2_O to obtain the target V_T_. Inspiratory time (Ti) was initially set at 0.4 s but was adjusted during the experiment to achieve the targeted V_T_. After 15 min lambs were ventilated for a further 1 h and 45 min using volume-guarantee to deliver V_T_ of 7 ml/kg, with PEEP of 5 cmH_2_O, inspiratory time of 0.5 s and expiratory time of 0.5 s. Total ventilation time was 120 min. The fraction of inspired oxygen (FiO_2_) was initially set at 40% but was adjusted during the experiment to maintain arterial blood oxygen saturation at 85–95%. This ventilation regimen elicits reproducible lung inflammation and injury in preterm lambs^11,27^.

#### Treatment

Prior to delivery, lambs were randomized to receive either intra-tracheal administration of 3% PS50G in saline (2 ml) (VENT+NANO; n = 6) via the endotracheal tube or no treatment (VENT; n = 5). A single 30-s sustained inflation was given to lambs in the VENT+NANO and VENT groups to help aerate the lungs^28^, with the aim of uniformly distributing the nanoparticles.

Arterial blood samples were collected every 5 min for the first 15 min of ventilation, and then every 15 min until the completion of the experiment, for measurement of arterial pH, PCO_2_, PO_2_ and SO_2_ (Fig. 2. 1; ABL30; Radiometer, Copenhagen, Denmark). Expiratory time was adjusted to maintain PaCO_2_ between 45–60 mmHg.

Arterial pressure, carotid blood flow, blood oxygen saturation, heart rate and ventilator outputs, including V_T_, mean airway pressure, PIP, and PEEP were monitored and recorded continually throughout the study (Powerlab; ADInstruments, Castle Hill, NSW, Australia).

After ventilation, lambs were humanely killed (sodium pentobarbitone; >100 mg/kg IV). Tissue samples from all groups were collected immediately (see below).

#### Lung tissue collection for histological analysis

The lungs were dissected from the chest, and the upper lobe of the right lung was fixed by airway instillation of 10% formalin at 20 cmH_2_O. After processing, 3 random samples were embedded in paraffin. Ten consecutive 5-µm sections were cut from each sample. Three of the 5-µm sections (each 25 µm apart), were stained with haematoxylin and eosin (H&E) and scored for gross lung injury based on airway wall thickness, epithelial sloughing, and immune and red blood cells within the tissue and airways^29^. Monocytes/macrophage counts were performed on 3 non-overlapping fields of view from 3 lung tissue sections, immunohistochemically stained with an antibody to CD163 (SM2160P, Acris Antibodies, Germany).

All histological assessments were made by an investigator blinded to group assignment of the sections.

#### Lung tissue collection for gene expression analysis

The lower lobe of the right lung was chopped into small pieces, which were immediately frozen in liquid nitrogen. Subsequently, approximately 600 mg of the frozen tissue was weighed and homogenized (Ultra-Turraz T-25; Janke & Kunkel, IKA-Laboritechnik, Germany) for 30 s in 15 ml of lysis buffer (Buffer RLT, Qiagen, Australia) with 150 µl of β-mercaptoethanol (Sigma-Aldrich, Australia). Total RNA was extracted from homogenized samples (RNeasy Midi Kit, Qiagen) and was reverse-transcribed into cDNA (SuperScript III reverse transcriptase, Invitrogen). Genes of interest were measured by qRT-PCR (Applied Biosystems 7900 HT Fast Real-Time PCR system). Relative mRNA levels (normalized to that of the housekeeping gene *1*8* S* using the cycle threshold (C_T_) method of analysis) of the pro-inflammatory cytokines interleukin (*IL*) -*1β*, -*6* and -*8*, anti-inflammatory cytokine *IL-10* and early injury genes *EGR1*, *CYR61*, and *CTGF* was quantified (primer sequences and PCR conditions are shown in Table 1). The expression of all genes is expressed relative to the mean of the UVC group.

**Table 1:** Primer sequences and polymerase chain reaction conditions.

Gene	Species	Genbank Accession#	Forward primer sequence: 5′-3′	Reverse primer sequence: 5′-3′	Annealing temperature	[cDNA]	[primer]
*18 s*	Rat	NR_003286	GTAACCCGTTGAACCCCATT	CCATCCAATCGGTAGTAGCG	60 °C	150 ng/µl	10 ng/µl
*IL-10*	Sheep	NM_001009327	GCTGTCATCGTTTTCTGCCC	CTCTCTTCACCTGCTCCACC	60 °C	150 ng/µl	10 ng/µl
*IL-8*	Sheep	NM_001009401	CCTCAGTAAAGATGCCAATGA	TGACAACCCTACACCAGACC	60 °C	150 ng/µl	10 ng/µl
*IL-6*	Sheep	NM_001009392	CGCAAAGGTTATCATCATCC	CCCAGGAACTACCACAATCA	60 °C	150 ng/µl	10 ng/µl
*IL-1β*	Sheep	NM_001009465	CGATGAGCTTCTGTGTGATG	CTGTGAGAGGAGGTGGAGAG	60 °C	150 ng/µl	10 ng/µl
*CYR61*	Sheep	DQ239628	ATCGTCCAAACAACTTCGTG	GGTAACGCGTGTGGAGATAC	60 °C	150 ng/µl	10 ng/µl
*CTGF*	Sheep	DQ239672	TATAGCTCCAGCGACAGCTC	ACGAACTTGACTCAGCCTCA	60 °C	150 ng/µl	10 ng/µl
*EGR1*	Sheep	DQ239634	AGGGTCACTGTGGAAGGTC	GCAGCTGAAGTCAAAGGAA	60 °C	150 ng/µl	10 ng/µl

### Statistical Analysis

Data are presented as mean ± SEM unless otherwise stated. The change in BAL differential cell counts between the second (baseline) and third HDM challenge (after saline or nanoparticle treatment) were compared by t-tests (ANOVA: Prism 7 for Mac OS X, GraphPad Software, USA). Serial physiological data from preterm lambs were divided into 2 separate epochs: the initial 15 min of ventilation, and the subsequent 1 h 45 min. Each epoch was analysed separately because the method of ventilation was different. Comparisons between groups were made using 2-way repeated measures ANOVA, and the Holm-Sidak post-hoc test was used to compare between groups and/or time-points (SigmaPlot, Systat Software Inc, USA). Variables measured at single time-points in all 3 groups of lambs (birth weight, umbilical arterial blood gases, tissue measurements) were compared between groups using ANOVA or Kruskal-Wallis test, as appropriate, with Holm-Sidak or Dunn’s multiple comparisons test, respectively (Prism). Histological variables were compared between VENT and VENT+NANO groups using unpaired t-test (Prism).

## Results

### Effect of nanoparticles on allergic airway inflammation in sheep

**Figure 1:**
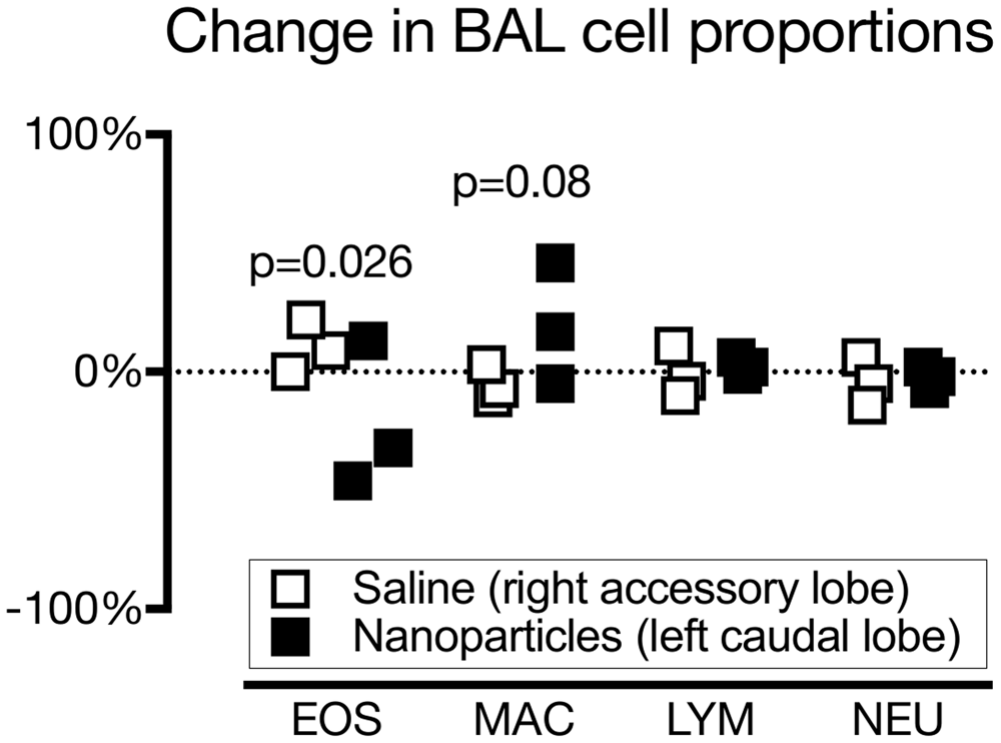
Effect of nanoparticle treatment on inflammatory cell recruitment at 48 h post-HDM challenge in response to airway challenge with house dust mite antigen in sensitized ewes. The change in the proportion of eosinophils (EOS) at 48 h post-HDM challenge, relative to baseline, was lower in bronchoalveolar lavage (BAL) samples from the nanoparticle-treated left caudal lobe (black squares) than the saline-treated right accessory lobe (white squares) of the lungs. Proportions of macrophages (MAC) tended higher after nanoparticle treatment. Lymphocytes (LYM) and neutrophil (NEU) proportions were not different between treatments.

The change in airway eosinophil recruitment in response to airway HDM challenge in sensitized sheep was lower in the left caudal lung lobe, where PS50G nanoparticles were administered, than in the saline-administered right accessory lobe (p = 0.026; Fig. 1). Corresponding to the decrease in eosinophil recruitment, there was a greater increase in % BAL macrophages in the PSG50-treated (left caudal) lung lobe compared to the saline-treated (right accessory) lobe (p = 0.08). Changes in the proportions of neutrophils and lymphocytes in BAL did not differ between treatments.

### Lamb details

All lambs in the VENT group were male. In the UVC and VENT+NANO groups, 20% and 14% of lambs, respectively, were male. Birth weights, birth order of twins and triplets, and umbilical arterial blood gas measurements were not different between groups (Table 2).

**Table 2:** Characteristics of lambs at birth.

	UVC	VENT	VENT+NANO
Number	5	5	6
Sex (% males)	20%	100%	14%
Birth Weight (kg)	2.7 ± 0.3	3.1 ± 0.2	2.6 ± 0.1
Lung Weight (g)	98.4 ± 13.7	108.6 ± 9.1	95.8 ± 8.4
Birth Order (Ratio first delivered: second or third delivered)	1:5	3:5	3:6
pHa	7.23 ± 0.03	7.19 ± 0.07	7.28 ± 0.01
PaO_2_ (mmHg)	32.9 ± 20.3	35.1 ± 9.3	25.8 ± 4.2
PaCO_2_ (mmHg)	53.7 ± 16.9	67.1 ± 7.4	54.9 ± 2.9

Weight and blood gas data are mean ± SEM.

### Ventilation

**Figure 2:**
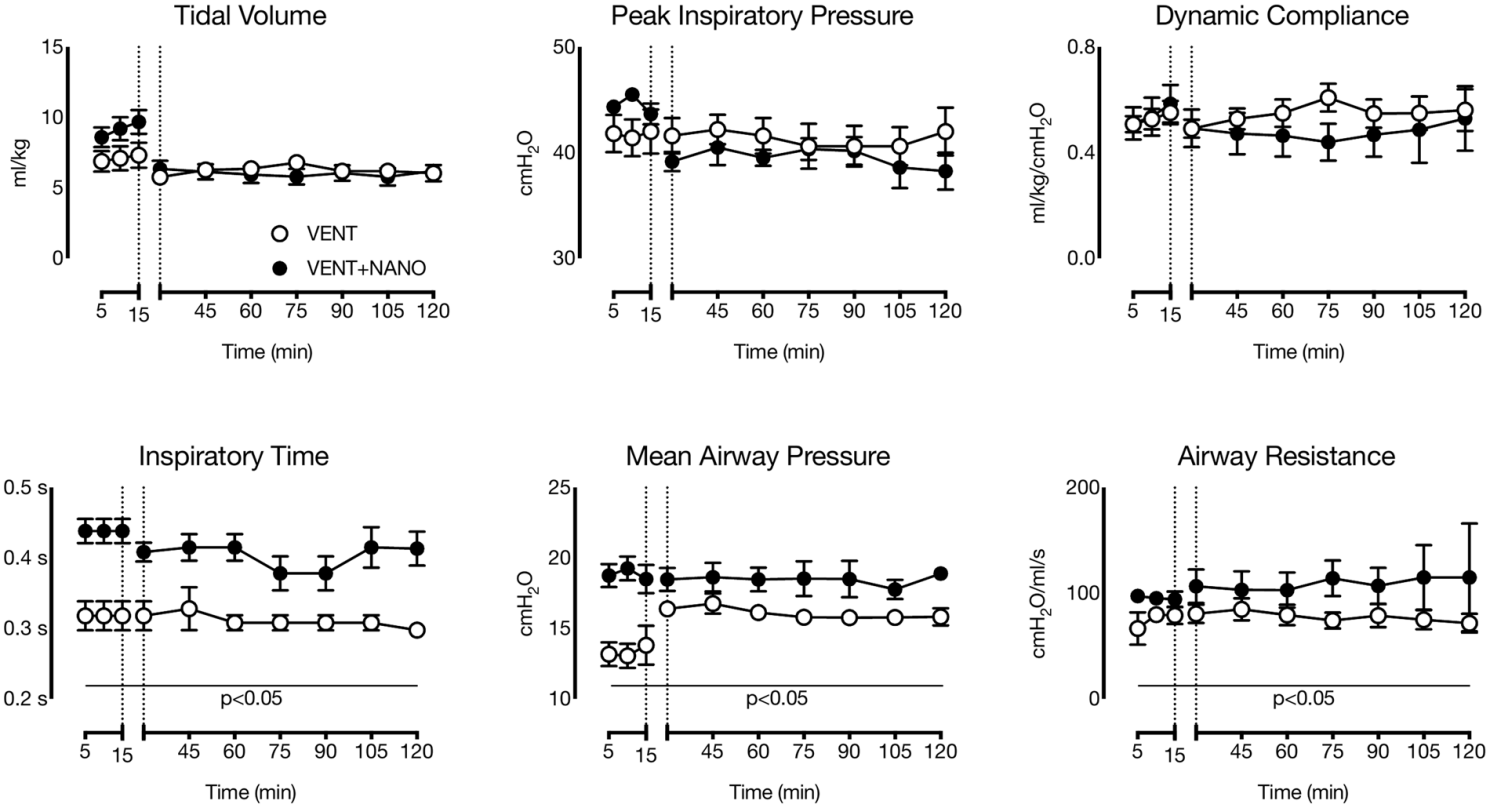
Respiratory mechanics in preterm lambs. Tidal volume, peak inspiratory pressure and dynamic compliance were not different between groups of preterm lambs treated with either nanoparticles (n = 6: VENT+NANO; black) or saline (n = 5: VENT; white). However, longer inspiratory times were required to achieve target tidal volumes, resulting in higher mean airway pressures in nanoparticle-treated lambs than in the saline group, likely a consequence of higher airway resistance in the nanoparticle-treated lambs. Horizontal lines show time periods over which values were different between groups (p < 0.05). Data are means ± SEM.

Indices of lung mechanics in preterm lambs are shown in Fig. 2. Peak inspiratory pressures (PIP) and tidal volumes (V_T_) were not different between groups, but mean airway pressure (P_AW_) was higher in the VENT+NANO group compared to the VENT group during both the initial 15 min of high V_T_ ventilation (*p* = 0.006) and the subsequent 1 h 45 min period (*p* < 0.0001). Inspiratory time was higher in the VENT+NANO group compared to the VENT group during the initial 15 min of high V_T_ ventilation (*p* = 0.055) and was also higher (*p* < 0.0001 during the subsequent 1 h 45 min period. Airway Resistance (R_AW_) was higher in the VENT+NANO group compared to the VENT group during both the initial 15 min (*p* = 0.01) and subsequent 1 h 45 min (*p* = 0.001) of ventilation. Dynamic Compliance (C_dyn_) was not different between groups and did not change throughout the 2 h ventilation period.

**Figure 3:**
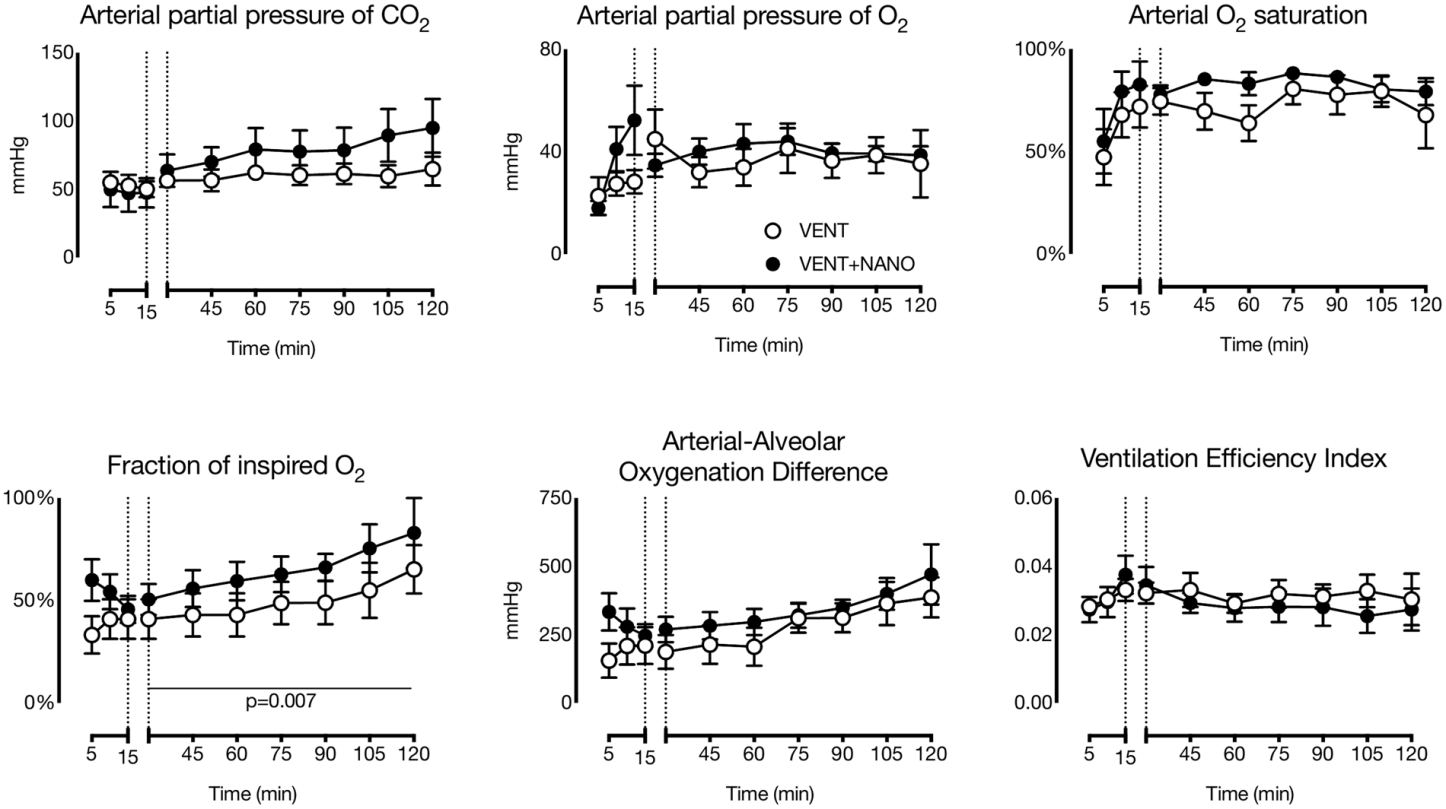
Respiratory gas exchange in preterm lambs. Blood gases were not different between groups of nanoparticle-treated (n = 6: VENT+NANO; black) or saline-treated (n = 5: VENT; white) preterm lambs. The inspired O_2_ content of VENT+NANO lambs was higher than the VENT group (horizontal line shows times at which FiO_2_ was different between groups), but the arterial-alveolar O_2_ difference and ventilation efficiency index were not different between groups. Data are means ± SEM.

Indices of lung gas exchange in preterm lambs are shown in Fig. 3. PaO_2_ and SaO_2_ were not different between groups and did not change significantly throughout ventilation. The fraction of inspired oxygen (FiO_2_) was not different during the initial 15 min of high V_T_ ventilation but was higher (p = 0.007) in the VENT+NANO group than the VENT group during the subsequent 1 h 45 min period of ventilation. The arterial-alveolar oxygen difference (AaDO_2_), PaO_2_ and ventilation efficiency index (VEI) were not different between groups and did not change significantly throughout the entire 2 h ventilation period.

### Lung inflammation and injury in preterm lambs

**Figure 4:**
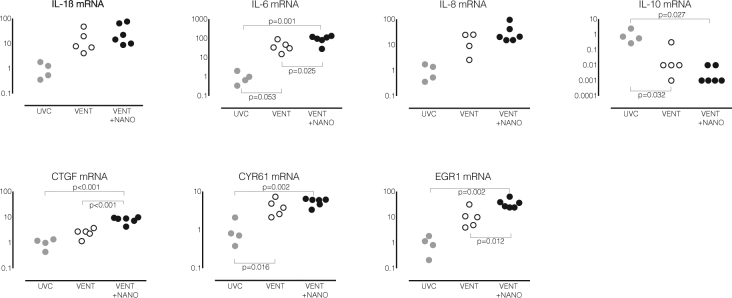
Expression of proinflammatory cytokine and early injury response genes. Messenger RNA (mRNA) levels for proinflammatory cytokines (interleukin (IL)-1ß, IL-6 and IL-8) and early injury response genes (cysteine-rich 61 (CYR61), connective tissue growth factor (CTGF), early growth response 1 (EGR1)) were higher, and for anti-inflammatory cytokine IL-10 were lower, in ventilated lambs treated with saline (VENT, white circles) or nanoparticles (VENT+NANO, black circles) than in unventilated controls (UVC, grey circles). Levels of mRNA for IL-6, CTGF and EGR1 were higher in the VENT+NANO group that the VENT group. Data are expressed relative to the mean of the UVC group.

Expression of proinflammatory cytokine and early injury response genes is shown in Fig. 4.

IL-6 mRNA levels were higher in VENT lambs compared to UVC (p = 0.0225), and were higher in VENT+NANO lambs than VENT (p = 0.0323) and UVC (p = 0.0012) group. IL-8 mRNA levels were higher in VENT and VENT+NANO lambs compared to UVC (p = 0.045 and p = 0.006 respectively). IL-1β mRNA levels were higher in VENT and VENT+NANO lambs compared to UVC but these differences were not statistically significant. IL-10 mRNA levels were lower in VENT (p = 0.0320) and VENT+NANO (p = 0.0269) lambs compared to UVC. CYR61 mRNA levels were higher in VENT+NANO (p = 0.0002) and VENT (p = 0.025) groups than UVC. CTGF mRNA levels were higher in VENT (p = 0.0194) and VENT+NANO (p = 0.0002) lambs than UVC. CTGF mRNA levels were higher in VENT+NANO lambs compared to VENT (p = 0.0004) and UVC lambs (p = 0.0002). EGR1 mRNA levels were higher in the VENT+NANO group compared to both UVC (p = 0.002) and VENT (p = 0.0185).

#### Histological analysis

**Figure 5:**
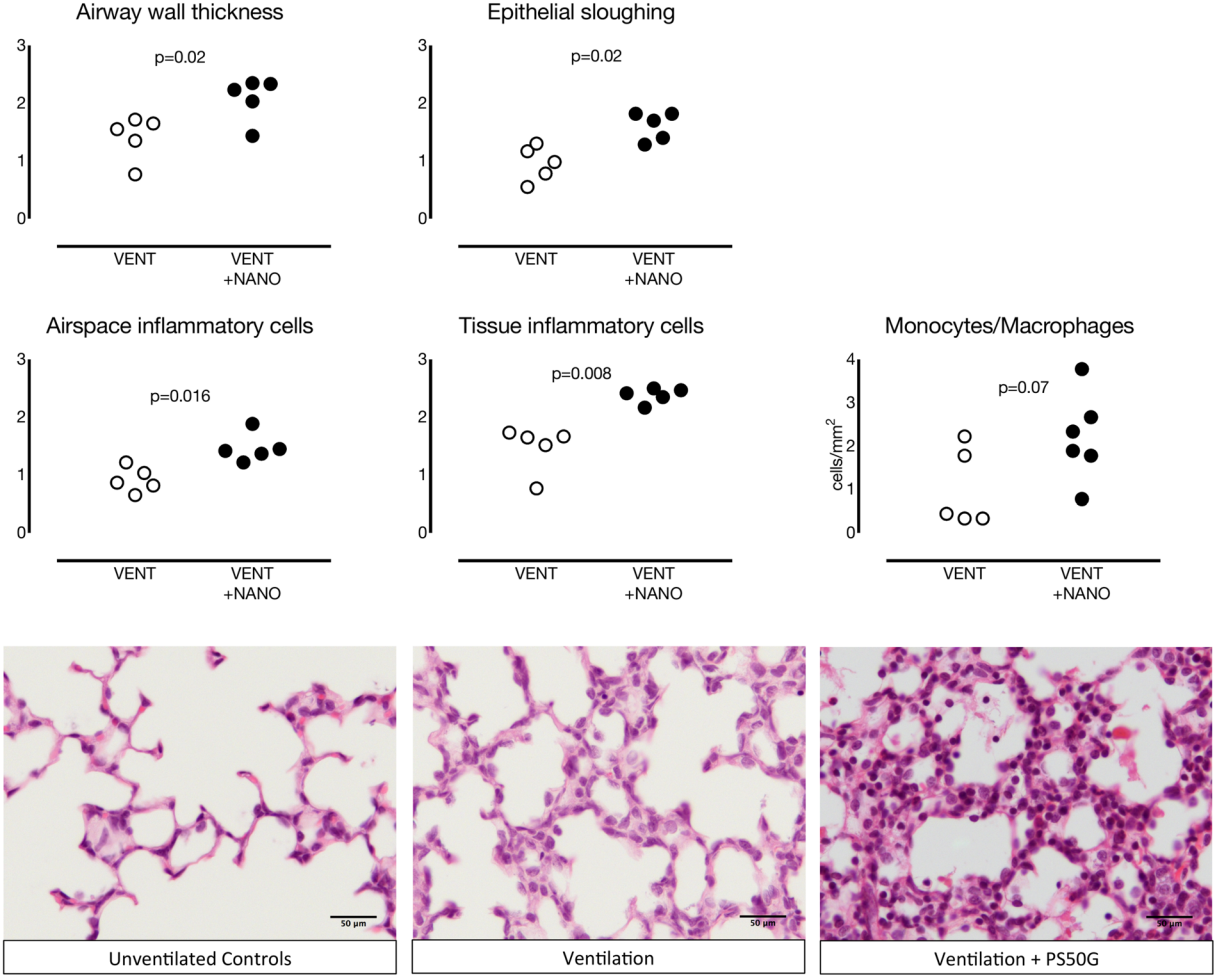
Histological assessment of lung injury in ventilated preterm lambs. Scores for airway wall thickness, epithelial sloughing, and airspace and tissue inflammatory cell counts were higher in nanoparticle-treated lambs (VENT+NANO, black circles) than in the saline-treated group (VENT, white circles). Monocyte/macrophage cell counts tended higher in VENT+NANO group than the VENT group. Representative images of hematoxylin and eosin staining in lung tissue of UVC, VENT and VENT+NANO. Scale bar = 50 μm.

Indices of histological assessment of lung inflammation and injury are shown in Fig. 5.

Histological scoring of airway wall thickness, inflammatory cell infiltration of the tissue and airways, and epithelial sloughing was greater in the VENT+NANO group than the VENT group (*p* = 0.05, *p* < 0.001, *p* = 0.001 and *p* = 0.001 respectively). Histological assessments of haemorrhage in the tissue and airways were not different between groups. The number of macrophages/monocytes in fetal lung tissue were higher in the VENT+NANO group than UVC (*p* = *0.05*); numbers in the VENT group were intermediate.

## Discussion and Conclusion

The initiation of ventilation at birth can cause lung injury, subsequent initiation of an inflammatory cascade and ultimately result in aberrant lung development^12^. Our study demonstrates that nanoparticles, which can reduce inflammation in one context, can exacerbate lung injury and inflammation induced by ventilation in preterm lambs. Increased airway resistance, suggesting acute adverse physical effects of nanoparticle administration, accompanied the exacerbation of lung injury. We confirmed the anti-inflammatory effect of PS50G, demonstrated initially in a mouse model of asthma^17,21^, by showing similar results in our sheep asthma model. The mouse studies suggest that the uptake of PS50G by dendritic cells (DC) results in a decrease of DC function and numbers^17,21^. Dendritic cells act to induce a proinflammatory response by processing foreign material and presenting antigens to immune cells^17,30^. The prevention of downstream effects of DC by PS50G is the likely mechanism of inflammation suppression. Dendritic cells are not present at birth in sheep or humans and are triggered to develop postnatally^31,32^. Therefore, such an anti-inflammatory action of PS50G is unlikely in the preterm lambs’ lungs. We believe, in the context of our study, an inability to reduce inflammation and the accompanying increased injury may be attributed, at least in part, to the difference between the pulmonary immune systems of the preterm newborn lamb compared to the lungs of adult mice and sheep. We consider that immaturity of the preterm lungs likely limits PS50G clearance by DC.

The principle aim of our investigations in lambs was to determine whether PS50 nanoparticles would have any bioactivity preterm newborns. We reasoned that the relative deficiency of pulmonary antigen presenting cells in preterm newborn lambs would limit the ability of PS50G nanoparticles to act through the same mechanism as they likely do in adult models of asthma. We opted for a high dose, which we considered most likely to have an effect. Having shown a biological effect of PS50G nanoparticles in preterm lambs, albeit a deleterious one, bioactivity is confirmed in this context. Future experiments are now justified to determine the effects of lower doses. Our work clearly shows caution is required around the use of nanoparticles in perinatal subjects.

Our study in preterm lambs used a ventilation strategy known to result in lung injury and inflammation, with initial delivery of high tidal volumes^11,33^. Stretch-induced injury of cells in the airways and lung parenchyma is a major contributor to neonatal lung injury and the pathogenesis of BPD^7^. In our experiment the tidal volumes delivered to ventilated lambs were not different between PS50G-treated and untreated groups. Thus, differences in stretch-induced injury are unlikely to be responsible for the differences in lung injury and inflammation between groups.

Our groups of preterm lambs had different proportions of male and female subjects. We are unaware of data showing differences in lung injury responses to ventilation in male and female preterm newborns, over a time frame like that in our study. De Matteo *et al*. recently showed no sex difference in lung injury of preterm lambs, as indicated by the presence of protein in bronchoalveolar lavage fluid 8 hours after birth^34^, and we showed that the cardiopulmonary hemodynamic transition at birth is not different between male and female preterm lambs^35^. Thus, the differences we observed between groups are unlikely confounded by sex of the subjects.

Although peak inspiratory pressures and tidal volumes were equivalent between groups, we observed evidence of impaired airway function in preterm lambs treated with PS50G. These lambs required longer inspiratory times to generate tidal volume, resulting in greater mean airway pressures (even though PIP was equivalent), consistent with the measured increase in airway resistance in these lambs. We consider this increase in airway resistance is likely the result of small airway blockage by the nanoparticles at the high dosage used in this study.

We believe the histological evidence of lung injury observed in our study is a direct consequence of PS50G nanoparticles in the lungs, rather than the changes in lung function we observed. Pulmonary exposure to particulate matter results in oxidative stress, resulting in subsequent promotion of a pro-inflammatory response, thus contributing toward chronic lung disease^36,37^. It is known that nanoparticles can induce greater responses than larger micron-sized particles^36^.

We observe an up-regulation of pro-inflammatory mediators in response to PS50G administration in preterm lambs. However, the 2-h timeframe of our experiment is too short for the increased inflammatory cascade to result in substantial morphological changes to the lung tissue. Mechanical ventilation of preterm lambs is known to increase pulmonary monocyte recruitment and differentiation of these cells to pulmonary macrophages^12^. PS50G nanoparticles more-than doubled pulmonary monocyte/macrophage recruitment in preterm lambs but this increase was not statistically significant. We consider that differences in the proportions of monocytes/macrophages in PSG50-treated versus untreated lung lobes in asthmatic adult sheep are likely the consequence of the reduction in eosinophils. We did not conduct total cell counts on bronchoalveolar lavage fluid samples from these sheep, so we cannot determine total monocyte/macrophage numbers.

We believe the increase in histological signs of lung injury seen in the PS50G group is due to an increase in mechanical stress caused by PS50G. Atelectrauma may have occurred from potential interference with surfactant resulting in an increase in surface tension. Furthermore, blockage within the airways may have resulted in excessive volume delivered to other lung regions, thereby resulting in volutrauma and barotrauma. We believe that high dose of PS50G further increased physical injury resultant from injurious ventilation, resulting in the observed changes to lung morphology. Our principle aim was to determine whether PS50 nanoparticles would have any bioactivity in ventilated preterm lambs. We reasoned that the relative deficiency of pulmonary antigen presenting cells in preterm newborn lambs would limit the ability of PS50G nanoparticles to act through the same mechanism as they likely do in adult models of asthma. We opted for a high dose, which we considered most likely to have an effect. Having shown a biological effect of PS50G nanoparticles in preterm lambs, albeit a deleterious one, bioactivity is confirmed in this context. Future experiments are now justified to determine the effects of lower doses. Our work clearly shows caution is required around the use of nanoparticles in perinatal subjects.

There are a number of potential approaches to inhibit pulmonary inflammation and injury, in addition to use of either inert^17^ or drug-conjugated nanoparticles^38–41^. Corticosteroids have long been used to inhibit pulmonary inflammation, and have benefit in reducing lung inflammation and chronic lung disease in preterm infants^42–44^; the challenge is identification of an effective but safe dose of these powerful anti-inflammatory agents, which have profound effects on development^44^. It is possible that inhaled corticosteroids may have utility in this respect. More precise anti-inflammatory therapies may also be able to reduce lung inflammation in preterm neonates. The TNF antagonist Etanercept^45^ and the interleukin-1 receptor antagonist Anakinra^46^ reduce lung inflammation and prevent consequent abnormal lung development in mouse models of bronchopulmonary dysplasia. Anti-interleukin 4Rα reduces inflammation in a mouse asthma model^41^. Other anti-inflammatory agents, which target the inflammasome^47^ or oxidative stress^48^ are effective in experimental settings. Stem cells and other cell therapies likely act via immunomodulatory mechanisms^49^.

## Conclusions

Our study shows that high dose of PS50G nanoparticles can exacerbate newborn lung inflammation and injury in response to mechanical ventilation. This is despite a demonstrated anti-inflammatory effect of PS50G nanoparticles in mouse and sheep models of allergic asthma. Our study suggests a cautious approach is required to the introduction of nanoparticle-based therapies in newborns.
